# The Goal of Adequate Nutrition: Can It Be Made Affordable, Sustainable, and Universal?

**DOI:** 10.3390/foods5040082

**Published:** 2016-11-30

**Authors:** Ian McFarlane

**Affiliations:** School of Agriculture, Policy and Development, University of Reading, Reading RG6 6AR, UK; i.d.mcfarlane@reading.ac.uk; Tel.: +44-(0)778-635-3485

**Keywords:** nutrition, linear programming, supply chain, agricultural economics, development, climate change, E210, E230, I150

## Abstract

Until about 1900, large proportions of the world population endured hunger and poverty. The 20th century saw world population increase from 1.6 to 6.1 billion, accompanied and to some extent made possible by rapid improvements in health standards and food supply, with associated advances in agricultural and nutrition sciences. In this paper, I use the application of linear programming (LP) in preparation of rations for farm animals to illustrate a method of calculating the lowest cost of a human diet selected from locally available food items, constrained to provide recommended levels of food energy and nutrients; then, to find a realistic minimum cost, I apply the further constraint that the main sources of food energy in the costed diet are weighted in proportion to the actual reported consumption of food items in that area. Worldwide variations in dietary preferences raise the issue as to the sustainability of popular dietary regimes, and the paper reviews the factors associated with satisfying requirements for adequate nutrition within those regimes. The ultimate physical constraints on food supply are described, together with the ways in which climate change may affect those constraints. During the 20th century, food supply increased sufficiently in most areas to keep pace with the rapid increase in world population. Many challenges will need to be overcome if food supply is to continue to meet demand, and those challenges are made more severe by rising expectations of quality of life in the developing world, as well as by the impacts of climate change on agriculture and aquaculture.

## 1. Introduction

Throughout history, until about 100 years ago, hunger and poverty were almost synonymous: a large proportion of the world population were poor, and whatever resources were available to the poor had to be allocated to obtaining food sufficient for survival.

**Figure 1:**
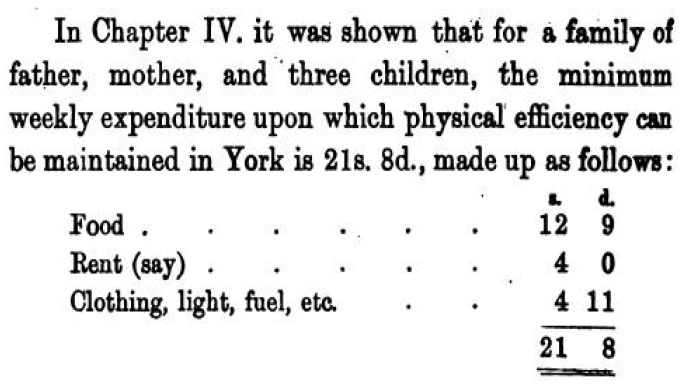
The poverty line in York, UK (from ref. [1]).

As societies became industrialised, a wider range of resources became available, but food remained the prime requirement for the poor. When Seebohm Rowntree published the results of his 1899 survey of households in York, UK [1] he defined ‘the poverty line’ as ‘minimum necessary expenditure for maintenance of merely physical health’. He first established data that set the level below which he considered a family to be in ‘primary’ poverty (Figure 1).

Rowntree found that almost exactly 10 percent of the population of York were living below the poverty line. He noted a generally low standard of health among the poor, and was able to attribute that to “inadequate nutrition of the poorer sections of the labouring classes”.

During the 20th century, living standards improved greatly. Throughout the developed world there were notable advances in food and agricultural sciences, in land management, in macro- and micro-economics and in understanding of sustainability of physical resources. In the year 2000, the United Nations Millennium Declaration defined eight Millennium Development Goals (MDGs) of which the first was:

MDG 1: Eradicate extreme hunger and poverty

The MDGs were extended in 2015 to 17 new Sustainable Development Goals (SDGs) as part of the 2030 Agenda for Sustainable Development. The first two SDGs recognised that poverty and hunger were no longer inseparable. They are:

SDG 1: No poverty
SDG 2: Zero hunger.

There are also specific goals for:

- Good health and well-being (SDG 3)
- Clean water and sanitation (SDG 6)
- Affordable and clean energy (SDG 7)
- Sustainable cities and communities (SDG 11)
- Responsible consumption and production (SDG 12)
- Climate action (SDG 13)
- Life below water (SDG 14)
- Life on land (SDG 15)

together with economic and political goals. These goals clearly involve multiple and overlapping disciplines.

Regarding agriculture, and observing factors that determine whether a particular country or global region can access sufficient food and nutrition for its population, Beachy [2] found that specific government policies, the quality of natural resources, market access, and international trade are involved in the many components of food security; a key determining factor is the complex set of policies that determine whether or not agriculture is a country priority. Beachy concluded that investment in research leads to increased agricultural productivity, and that other biological sciences and engineering sciences also have positive impacts in agriculture. As demands of a growing population and an expanding bioeconomy place greater expectation on agriculture and agro-ecosystems, Beachy considered that it is critically important that commitments for increased funding be made, country by country, and on a global scale.

The livestock sectors of agriculture, together with aquaculture, demand an economic tool for feed formulation. Advances in computing made it possible to use the method of linear programming (LP) to calculate the least-cost of rations purchased for farm-fed animals [3]. In feed formulation for animals and fish, the consumers have little opportunity to express any taste preference other than outright rejection. Darmon, Ferguson, and Briend [4] made use of LP to predict the food choices a rational human individual would make to reduce his or her food budget, while retaining a diet as close as possible to the food choice of the average population. Their results indicated that a simple cost constraint can decrease the nutrient densities of diets and influence food selection in ways that reproduce the food intake patterns observed among low socioeconomic groups. Availability of nutrients is often dependent on transportation and storage of food items, with significant costs which must be taken into account, plus allowance for spoilage. Food chain costs are also often subject to import tariffs, and in some cases these are offset by government subsidies.

Concerning human nutrition, Truswell and Mann [5] commented that the study of food habits overlaps with psychology, anthropology, sociology, and economics. Food habits vary widely, and many governments issue dietary recommendations that take note of national preferences. The Food and Agriculture Organisation of the United Nations (FAO) assists Member Countries to develop, revise, and implement food-based dietary guidelines; the FAO noted that more than 100 countries worldwide have developed dietary guidelines that are adapted to their nutrition situation, food availability, culinary cultures, and eating habits.

Fattore and Agostini [6] noted that wellbeing in a broad sense includes and goes beyond health effects, requiring collaboration between research programs in psychology, economics, and sociology.

The object of this paper is to review progress towards coordinating many aspects of development in disciplines aimed at improved quality of life. The paper is organised as follows. Section 2 examines progress in dietary guidelines and recommended daily intake (RDI). Section 3 uses livestock nutrition to introduce the concept of economic tools to optimise feed formulation, and explains the relevance of those tools to human nutrition. Diets for three case study examples are proposed, and costed according to specific criteria. Section 4 considers in some detail the worldwide variation in dietary preferences, and the progress that has been made towards ensuring that healthy options can be sustained indefinitely for every dietary regime. Section 5 examines the constraints that ultimately restrict food supply, including the ways in which climate change may modify those constraints. The challenges to maintenance of food supply to meet the increasing expectation of food choices within a still expanding world population, with the added problems associated with climate change and sustainability requirements, are summarised in Section 6.

## 2. Trends in Dietary Recommendations

### 2.1. Developments in Nutrition Science

Nutrition science in its modern form dates from the mid-19th century (Cannon, 2005 [7]), and has received particular attention from food shortages and rationing of food supply during the wars of 1914–1918 and 1939–1945. Dietary reference intakes (DRI), recommended dietary allowances (RDA), and recommended nutrient intakes (RNIs) have since been used as the basis for national nutrition monitoring and for intervention programs. In addition to carbohydrate, lipid, and protein intake, guidelines include provision of iron, calcium, and vitamins, together with trace elements and other biologically active substances.

An overview of the process for defining DRI was given by Yates [8]. She noted that DRIs represent the best scientific perspectives regarding what should be the basis for nutrition and public health policy related to foods and supplements. Yates further commented that new scientific results can require changes to be made. Thus, for vitamin B-12, as for many nutrients, absorption is dependent on available transport mechanisms and the load put on the gastric system. It is often the different assumptions related to bioavailability that alter the reference intakes, rather than the basic science from which the recommendation is derived.

Truswell (in ref. [5]) noted that the earliest set of recommendations that included micronutrients was issued by the League of Nations in 1937. The United States Department of Agriculture (USDA) first issued a set of RDA in 1943, subsequently revised many times. The current recommendations for the USA can be found set out in formats for various applications in a section of the USDA website. Truswell distinguished between dietary goals, aimed at reductions in degenerative diseases, and dietary guidelines, which assist individuals in making choices among food options.

### 2.2. Range of Daily Intake Recommendations

As mentioned in the Introduction, the FAO has assisted many countries worldwide to issue dietary guidelines that are adapted to their nutrition situation, food availability, culinary cultures, and eating habits. In wealthy nations, governments provide detailed scientific advice, such as was made available in UK in 1991 [9], but in the 25 years since that publication, encouraged by the FAO, the UK and other national governments have condensed relevant information into shorter formats more easily assimilated by consumers. For example, a page on the UK National Health Service website [10] explains the reference values printed on food packaging for consumers:

The term “reference intakes” (or “RIs”) has replaced “guideline daily amounts” (“GDAs”), which used to appear on food labels. But the basic principle behind these two terms is the same.

…

Unless the label says otherwise, RI values are based on an average-sized woman doing an average amount of physical activity. This is to reduce the risk of people with lower energy requirements eating too much, as well as to provide clear and consistent information on labels.

As part of a healthy balanced diet, an adult’s reference intakes (“RIs”) for a day are:

 Energy: 8400 kJ/2000 kcal

 Total fat: 70 g

 Saturates: 20 g

 Carbohydrate: 260 g

 Total sugars: 90 g

 Protein: 50 g

 Salt: 6 g

The RI for total sugars includes sugars from milk and sugars contained in fruit, as well as added sugar.

In another example, from the Netherlands, full scientific information is provided in a formal report [11] with the following accompanying summary [12]:

- Follow a dietary pattern that involves eating more plant-based and less animal-based food
- Eat at least 200 g of vegetables and at least 200 g of fruit daily
- Eat at least 90 g of brown bread, wholemeal bread, or other wholegrain products daily
- Eat legumes weekly
- Eat at least 15 g of unsalted nuts daily
- Take a few portions of dairy produce daily, including milk or yogurt
- Eat one serving of fish weekly, preferably oily fish
- Drink three cups of tea daily
- Replace refined cereal products with whole-grain products
- Replace butter, hard margarines, and cooking fats with soft margarines, liquid cooking fats, and vegetable oils
- Replace unfiltered coffee with filtered coffee
- Limit the consumption of red meat, particularly processed meat
- Minimise consumption of sugar-containing beverages
- Do not drink alcohol or no more than one glass daily
- Limit salt intake to 6 g daily
- Nutrient supplements are not needed, except for specific groups

Kromhout et al. (in ref. [12]) explained that these Dutch dietary guidelines are based on 29 systematic reviews of English language meta-analyses summarizing randomized controlled trials, and cohort studies on nutrients, foods and food patterns and the risk of 10 major diseases.

For less developed countries, the FAO has assisted preparation of easily assimilated guides that can be compiled and approved without costly preliminary research. As a further example of guidelines, those for the population of the Seychelles [13] are stated as follows:

- Eat a variety of different foods in the proportion shown in the Seychelles Food Guide
- Consume at least five portions of fruit and vegetables every day
- Replace rice with wholegrains and other high fibre starchy foods at least three times a week
- Eat fish on at least five days a week
- Eat pulses (peas, beans, and lentils) at least four times a week
- Reduce the amount of cooking oil, fats, and fatty foods
- Remove fats on meat before cooking
- Limit the frying of foods to only once a week
- Consume sugar, sugary foods, and sugary drinks in minimal amounts
- Include three portions of milk and milk products in your diet every day
- Use salt and salty foods in small amounts
- Drink at least eight glasses of water every day
- If you drink alcohol, do not exceed the recommended amounts
- Maintain a reasonable body weight by exercising for 30 min every day
- Breastfeed your child exclusively up to six months
- Practice good hygiene when handling food.

These 2016 examples, for The Netherlands, a developed country, and the Seychelles, relatively undeveloped, are similar in being more wide ranging and less prescriptive than the 1991 ‘reference intakes’ for the UK.

## 3. Economics of Nutrition

### 3.1. Livestock Feed

The nutritional needs of farm animals with respect to energy, protein, minerals, and vitamins have been essential input data in systems for minimising feed costs in livestock farming. The determination of cost of meeting the feeding specifications for cattle, pig, and poultry production is carried out using well-established LP calculation methods; this provides a useful starting point for diet costing under the more complex constraints of feeding the human population. Rehman and Romero [14] demonstrated multiple-criteria decision-making techniques in livestock ration formulation, including goal programming and its variants such as weighted and lexicographic approaches and multiple-objective programming.

In a review of livestock production trends, Thornton [15] noted that increases in livestock productivity have been driven mostly by developments in breeding, nutrition, and animal health. Various requirement determination systems exist to assess the nutritional and productive consequences of different feeds for the animal once intake was known. Arthur, Archer, and Herd [16] assessed the efficiency of feed utilisation in beef cattle in Australia. They found that genetic variation in feed efficiency exists in Australian beef herds, that feed efficiency is moderately heritable and that the potential exists to reduce the cost of beef production through selection for efficient cattle. Using the example of pig production, Burlacu and Nitu [17] showed how a multi-objective fractional programming model can be used to extend LP to include non-linear effects; Kyriazakis [18] reported net efficiency of energy and nutrient utilisation and partitioning of scarce resources within productive functions with reference to pigs and poultry.

Ghosh et al. [19] pointed out a shortcoming of LP in that animals not only require minimum quantities of protein, energy, and minerals but also that excess feeding has to be curbed to prevent excess excretion of nitrogen, minerals, and emission of methane; they showed that stochastic programming provides a more appropriate approach to feed formulation, and introduced a non-linear framework when a fractional objective is used, expressed as a ratio of two functions.

### 3.2. Cost of Applying Human Nutrition Standards

Riches [20] explored the relationship between hunger, food security, and welfare policies in Australia, Canada, New Zealand, UK, and the USA. Noting that there was sufficient food production worldwide in 1995 to provide everyone in the world with 2500 food calories per day, Riches commented that case studies in the ‘first world’ countries he studied indicated that public action should focus on developing national plans for nutrition and food security. Dowler and Dobson [21] reported that low-income households in UK are very skilled at budgeting, and food is often the only flexible item of household expenditure; in 1991 households in the bottom quintile spent UK £21.07 a week on food. The affordability of a nutritious diet for households in Toronto, Canada, that were supported by welfare in the year 1999 was assessed by Vozoris et al. [22] who found that a single-parent household subsisting on welfare support spent CAN $247.78 a week on food out of total weekly outgoings of CAN $1,367.65. Williams et al. [23] calculated similar weekly household expenditure by a typical Australian family in the period 2000–2007 to purchase a food basket for a family of five; they used the ‘Illawarra Healthy Food Basket (IHFB)’ of 57 items selected to meet their nutritional requirements, and found that a reference Australia family relying on welfare payments would need to spend just under 30% of the household income to purchase the IHFB.

Darmon et al. [24] calculated the impact of a cost constraint on the food choices required to provide a nutritionally adequate diet for French women in 2005; daily diets fulfilling both palatability and nutritional constraints were modeled in linear programming, using different cost constraint levels. For each modeled diet, total departure from an observed French population’s average food group pattern (“mean observed diet”) was minimized. To achieve the nutritional recommendations without a cost constraint, the modeled diet provided more energy from fish, fresh fruits, and green vegetables and less energy from animal fats and cheese than the “mean observed diet”. Introducing and strengthening a cost constraint decreased the energy provided by meat, fresh vegetables, fresh fruits, vegetable fat, and yogurts and increased the energy from processed meat, eggs, offal, and milk. For the lowest cost diet (€3.18/day), marked changes from the “mean observed diet” were required, including a reduction in the amount of energy from fresh fruits (−85%) and green vegetables (−70%), and an increase in the amount of energy from nuts, dried fruits, roots, legumes, and fruit juices. 

The U.S. Department of Agriculture (USDA) 2006 Thrifty Food Plan (TFP) [25] offered a useful framework for studying the cost of a nutritious diet. USDA generated the TFP by solving a constrained optimization problem, choosing a diet that was as similar as possible to the then current consumption pattern for low-income Americans. Wilde and Llobrera [26] explained that in the TFP framework, the goal is to choose a food plan which minimizes an objective function while simultaneously meeting a cost constraint, nutrition constraints, and other miscellaneous constraints.

### 3.3. Case Studies

Three countries were selected for case studies: Argentina, Bangladesh, and Canada. These case studies provide one example each from intermediate, low and high income countries ranked by 2011 GDP. I then selected alternative diets for each case study, based on the proportions in which foods were consumed in those countries, as reported in FAO Food Balance Sheets for 2011 [27] and associated prices for that year in local currency [28]. Set proportions of food intake were proposed for each country, each set to provide sufficient food energy of 10 MJ/cap/day with:

SET 1—food quantities in the proportions actually consumed in that country
SET 2—quantities adapted to approximate to a TFP
SET 3—quantities for sufficient nutrients at least cost.

Food items and prices, as reported to FAO for year 2011, are shown in Table 1. The energy, protein, and nutrient content of food items related to the FAO codes listed in Table 1 are shown in Table 2. The targets for energy, protein, and nutrient intake were defined using the UK 1991 dietary reference values as shown in Table 3. We estimated the cost of local supply of readily available food items on the basis of FAO data shown in Table 1, in each of 16 food categories, plus refined sugar.

I calculated the daily cost of a food basket per capita, for each SET of items for each country. For SET 3, I added the additional constraint that the basket contained wheat and cereal quantities approximately equal to the known daily intake used for SET 1, on the assumption that wheat and cereal supply chains and eating habits are well established in all three countries.

#### 3.3.1. Food Intake for Sufficient Energy

Results for food intake in each food category sufficient to provide 10 MJ/cap/day are shown in Table 4. Three sets of quantities were calculated for each case study:

- The first set represents g/day of a locally sourced item in each category, weighted in proportion to the consumption in that country of food items in that category in 2011
- The second set is calculated using weighting chosen to simulate the USDA TFP dietary recommendation
- The third set represents wheat and other cereal intake set approximately in proportion to the known daily intake calculated for the first set, with other quantities obtained using linear programming to yield at least the RNI of each nutrient in Table 3.

An estimate of the cost of a locally sourced example of food in each category (based on costs in local currency shown in Table 2) was used to calculate expenditure on each item in each set (shown in columns headed ‘LCU/day’ in Table 4). The sum total expenditure required is shown at the foot of each expenditure set in Table 4.

#### 3.3.2. Calculated Nutrient Intake

Combining the g/day intake with the protein and nutrient content of items in each food category based on data in Table 2, I calculated the nutrient content achieved in each intake set, with results shown in Table 5.

#### 3.3.3. Comment on Case Study Outcomes

The costs per person per day shown in Table 4 are consistent with the ‘dollar-a-day’ expenditure at bulk prices that has been used to define a ‘poverty line’, extensively reported and discussed over a number of years [31,32,33].

The USDA TFP intake pattern (SET 2), aimed to achieve an affordable diet of superior nutrient content, is only slightly more costly that the typical intake (SET 1) in Canada, but significantly more costly in Argentina and Bangladesh, perhaps because their normal eating patterns are unlike the eating preferences in North America.

The results obtained using partial linear programming (SET 3) suggest that it is feasible to include affordable items that are locally sourced to achieve a nutritious intake, at a cost slightly lower than SET 1 expenditure in Argentina and Canada, and only slightly higher in Bangladesh.

As to nutrient targets, the values in Table 5 suggest that:

- normal eating patterns are deficient in vitamin C in all three countries
- all other nutrients are readily available from a combination of local products
- the USDA TFP pattern achieves the same balance of nutrients in each country, with only slight variation in locally sourced item properties.

Any deficiency in vitamin C can be readily remedied without significant cost, as demonstrated by the results for SET 3.

Overall, the case studies demonstrate that energy, protein, and nutrient targets can be readily achieved at an affordable price from widely varying diets in countries at any stage of development.

## 4. Local and National Eating Patterns

### 4.1. Dietary Habits and Preferences Worldwide

LP models produce unrealistic diets, because they fail to capture consumers’ preferences. Economic constraints set bounds to food choices, but many other considerations affect dietary habits, including enjoyment—eating is a pleasurable experience, to the extent that self-imposed discipline is usually applied [34]. Irz et al. [35] identified as a “taste cost” the short term loss of hedonic rewards; they observed that standard dietary recommendations are poorly adopted in many countries, especially among disadvantaged people. This was perhaps mainly due to the cost that compliance imposes on consumers in terms of taste and convenience.

Overweight and obesity pose one of the biggest public health challenges for high, middle, and low income countries. In a multi-level analysis of low and middle income countries, Conklin et al. [36] assessed whether minimum wage is related to overweight or obesity prevalence, and found that the association of minimum wage with obesity was negative in low-income countries but positive in middle-income countries; by contrast there was a monotonic increase in the association between education and obesity in low-income countries; while in middle-income countries, the opposite was observed.

### 4.2. Sustainability Constraints

The concept of sustainable diets has been expressed by FAO [37] as “those diets with low environmental impacts which contribute to food and nutrition security and to healthy life for present and future generations.” Sustainable Crop Production Intensification (SCPI) is a strategic objective of FAO, to be achieved by providing technical and policy assistance in four areas:

- increasing productivity through improved use of resources to achieve higher yields
- enhancing sustainable crop protection through Integrated Pest Management
- managing biodiversity with soil, nutrient, and water management
- strengthening livelihoods within the value chain.

Auestad and Fulgoni [38] reviewed environmental and economic impacts of dietary patterns, including habitual eating patterns, nutritionally balanced diets, and a variety of different dietary scenarios. They compared 31 studies, mainly dealing with Europe and North America, addressing the climate impact of eating patterns in terms of land and agricultural capacity, primary energy use, and water use. They noted LP modeling results with and without acceptability constraints. Without constraints, diets could achieve 90% reductions in greenhouse gas (GHG) emissions, and 36% reductions with constraints that did not eliminate meat or dairy foods or increase the cost to the consumer.

Regarding agricultural GHG, Bennetzen et al. [39] calculated changes in emissions per unit of production in nine world regions, and found that intensive and industrialised systems show the lowest emissions per unit of agricultural production. Livestock production is a major source of emissions; Hyland et al. [40] found some limited opportunities for reducing emissions through efficiency gains, but emissions from dairy herds are likely to remain a cause for concern.

Soil-related challenges, including using soils and other natural resources sustainably, were assessed by Hurni et al. [41] who noted that the greatest needs and potentials lie in small-scale farming.

## 5. Long-Term Resource Constraints

### 5.1. Irrigation

Sustainable farming is neither practical nor possible in certain locations, where protecting water quality and promoting agricultural production may be incompatible. Doody et al. [42] examined approaches to prioritization and considered how catchment buffering capacity could be utilized. They reported that the buffering capacity of a system will eventually reach a threshold (saturation) level, defined as the point at which small changes in the inputs to a catchment cause a rapid change in the aquatic ecosystem, and observed that anthropogenic factors that lower catchment buffering capacity will also affect farmers’ adaptive capacity.

The greatest water stresses are associated with rice cultivation. Huang et al. [43] presented a comprehensive analysis of water used for food production in China over the period 1998–2010 based on modelling of agricultural water use coupled with national and provincial statistics. They reported a declining trend in national precipitation and internally renewable water resources, combined with existing water shortages and increasing competition for water from non-agricultural sectors. Crop water productivity (CWP) increased by 19.5% over the 13 years to 2010, but such productivity increases will be harder to achieve in the future. Cotton cultivation consumes a major proportion of the water available in the arid region of northwestern China, according to Shen et al. [44]. A concern is that in many of the ‘breadbasket’ provinces additional CWP gains may prove difficult. Huang et al. concluded that the historic efficiency gains give reason for optimism provided that there is continued investment in genetic improvement and innovation of farming systems.

### 5.2. Aquaculture

Aquaculture production has been expanding rapidly, and there is a large potential for further increases in fish supply. A report prepared by the International Food Policy Research Institute (IFPRI) for FAO and the World Bank (Msangi et al. [45]) stated that capture fisheries production increased from 69 million to 93 million tons in the period 1980–2010, while farmed fish production increased from 5 million to 63 million tons during the same period. Fish are low in saturated fats, carbohydrates, and cholesterol, and provide not only high-value protein but also vitamins, minerals, and polyunsaturated omega-3 fatty acids (PUFA). Many of the fishers and fish farmers in developing countries are smallholders. The IFPRI economic model predicted that total fish supply will increase to 186 million tons by 2030; within that total, China will account for 37 percent of total fish production (17 percent of capture production and 57 percent of aquaculture production). The IFPRI report included the prediction that aquaculture can adapt to climate change in such a way as to maintain production under various climate scenarios.

In a review published by an international team in 2016, Thilsted et al. [46] demonstrated that ecosystem-based management of capture fisheries can increase both fish stocks and biodiversity, and that the management of farmed fish systems also has potential for increasing productivity and species diversity. Within aquaculture, pond polyculture systems are a way of realising a mix of nutrient-rich small fish species and ‘cash-crop’ species for household consumption to maximize the use of input resources—similar to the principle of intercropping.

### 5.3. Pest Management

Factors likely to affect arable crop yields by 2050 were reviewed by Jaggard et al. [47], who noted that the expected atmospheric CO_2_ enrichment is likely to increase yields of most crops by approximately 13 percent. Competition from weeds will also be stronger, and pest pressures will be more severe as a result of global warming. Jaggard et al. considered that most weeds and airborne pests and diseases should remain controllable. However, large gaps will remain between achievable yields and those delivered by farmers. Soil borne pathogens are likely to be an increasing problem when warmer weather will increase their multiplication rates; crop rotation has long been the recommended strategy for managing such pathogens, but control is likely to need a transgenic approach to breeding for resistance.

### 5.4. Biotechnology

Enhanced nutrition obtained via biofortification has been achieved in a number of crops consumed by millions of people in Africa, Asia, and Latin America. Crops developed in the HarvestPlus program (www.harvestplus.org) include vitamin A-rich varieties of sweet potato, cassava, and maize, high-iron varieties of beans and pearl millet, and zinc-rich rice [48].

Mutagenesis (mutation crop breeding) has been in use since 1930, and FAO in 2014 documented more than 3200 officially released mutant varieties from 214 different plant species in more than 60 countries throughout the world. Belhaj et al. [49] commented that genome editing has emerged as an alternative to classical plant breeding as well as to the transgenic (GMO) methods that have found little acceptance in many countries. They pointed out that refinement of mutagenesis to make it more specific has enormous potential to improve crop plant performance. Ricroch and Hénard-Damave [50], in a review of progress in plant breeding, commented that R&D programs are flourishing in developing countries, boosted by the necessity to achieve food security while mitigating climate change impacts. A wide variety of plants are currently tested for their high yield despite biotic and abiotic stresses. Many plants with higher water or nitrogen use efficiency, tolerant to cold, salinity, or water submergence are being developed, together with biofortification in vitamins and metals.

The extensive role of reactive nitrogen (Nr) in agricultural systems was described in detail by Galloway et al. [51], who commented that although the ability to fix N on large scales is unquestionably a boon to humanity, in many developed nations, the products from N-intensive agricultural practices lead to unhealthy diets, whereas elsewhere a lack of synthetic fertilizers, combined with depleted soil nutrient reserves, directly contributes to widespread malnutrition. They concluded that:

- reducing Nr creation is both possible and of critical importance
- intervention is also needed in regions that do not have sufficient Nr to seek ways to increase food production while minimising environmental damage.

The regulation of biological nitrogen fixation by some bacteria has led to the suggestion that gene editing could be used to make fertiliser without the present scale of damage to the environment [52].

In another example of pushing back constraints on agricultural productivity, advances in genomics capabilities have led plant biologists to envisage the introduction of the C4 photosynthetic pathway into C3 crops such as rice and soybeans. Sage and Xin-Guang Zhu [53] observed that better understanding of the function of C4 photosynthesis provided new ways to improve existing C4 crops and bioenergy species, for example by creating varieties with ultra-high water and nitrogen use efficiencies, and that the main enzymes of the C4 metabolic cycle have already been engineered into various C3 plants.

## 6. Challenges to Maintenance of Food Security

Many difficulties stand in the way of the provision of a food supply sufficient to meet the increasing expectations of food choices within a still expanding world population, with added problems associated with climate change and sustainability requirements. Nutritionists and dieticians are well able to advise on healthy diets, and to show that a healthy diet is achievable in almost all diet preference regimes. The challenge is to provide a nutritious diet at an affordable price. Legumes (including peas and beans) and pulses (dried forms of legumes, such as lentils) have an important role in improving nutrition, with the advantage of being widely available in forms well adapted to local dietary preferences.

Food waste and spoilage post-harvest present a further challenge to food security. In a wide ranging 2010 review by an international team of scholars, Godfray et al. [54] reported that roughly 30% to 40% of food in both the developed and developing worlds is lost to waste. In the developing world, losses are mainly attributable to the absence of food-chain infrastructure and the lack of knowledge or investment in storage technologies on the farm. Even with rice grain, which can be stored readily, Godfray et al. observed that as much as one-third of the harvest in Southeast Asia can be lost after harvest to pests and spoilage. It will remain as a major challenge to reduce spoilage and losses post-harvest. New crops and traits are being developed in developing countries, often by research teams with joint public/private support. Various breeding techniques are very effective when used in combination. These are not alternatives to transgenesis, but serve to complement each other, and gene bank biodiversity is an essential contributing factor. Genetic diversity represents the heritable variation among plant species. Rao and Hodgkin [55] commented that in order to manage conserved germplasm better, there is also a need to understand the genetic diversity that is present in collections. This will help to develop better protocols for regeneration of germplasm seed. Through improved characterization and development of core collections based on genetic diversity information, it will be possible to exploit the available resources in more valuable ways.

Water availability for irrigation is an increasing cause for concern, directly and indirectly: mechanical pumps to irrigate crops have increased farm energy use, allowed larger water withdrawals, and contributed to aquifer depletion worldwide. As water tables drop, ever more powerful pumps have been introduced. More efficient irrigation systems—such as low-pressure and drip irrigation, and precision soil moisture testing—could reduce agricultural water and energy needs. Countering the historical trend toward more energy-intensive farm mechanization has been the adoption of conservation tillage methods. Soil quality is improved through this technique, while farm fuel use and irrigation needs are lowered.

There remain two main problems: the economic issue of providing a healthy diet for those with the lowest incomes, and the anxiety as to whether farming systems can continue to keep pace with food demand in a manner that is fully sustainable.

In this paper, I have reviewed progress in nutrition science and given examples of dissemination of dietary recommendations promulgated by national governments and by international bodies, in particular the United Nations FAO. I have described the use of linear programming to minimise diet cost, and still provide a diet that is palatable, culturally acceptable, and available to those with low income. Three case studies of countries with high, intermediate, and low GDP per capita, have been presented to support a conclusion that adequate nutrition can be readily achieved at affordable cost from widely varying diets in countries at any stage of development.

Regarding the sustainability of adequate nutrition, I have reviewed the constraints that may affect food supply under changing climate conditions. There are many complex problems yet to be solved, not least in the management of irrigation and of pest pressures. Agricultural science has a good record so far of dealing with such problems within economic constraints. There are grounds for optimism that current progress in biotechnology will provide farmers with the tools they will need to continue to meet the demand for healthy food for all sectors of human society in a sustainable manner.

## Figures and Tables

**Table 1:** Case studies: Food item consumption and local prices for the year 2011.

		Argentina		Bangladesh		Canada	
2011 GDP/cap in 2011 US $ [29]:		13,400		839		52,100	
	FAO						
Food Item	Code	kg/cap/year	ARS/kg	kg/cap/year	BDT/kg	kg/cap/year	CAN$/kg
Apples	2617	16.5	0.507	0.9	52.370	21.6	0.392
Bananas	2615	13.9	*7.150*	5.2	*35.280*	14.7	*0.590*
Barley	2513	18.9	*3.910*	0.0	*19.290*	174.8	*0.195*
Beans	2546	0.7	*9.200*	0.3	*14.870*	1.5	*0.697*
Bovine Meat	2731	55.1	7.810	1.3	195.310	29.7	2.161
Butter, Ghee	2740	0.6	*72.990*	0.2	*360.040*	2.8	*5.970*
Cassava	2532	4.7	*0.790*	0.8	*3.920*	1.0	*0.070*
Cocoa Beans	2633	0.9	*48.480*	0.0	*239.140*	2.6	*3.970*
Crustaceans	2765	0.3	*47.690*	1.2	*235.220*	5.8	*3.900*
Dates	2619	0.0	*24.800*	0.0	*122.310*	0.3	*2.030*
Demersal Fish	2762	2.1	*44.510*	0.6	*219.530*	5.1	*3.640*
Eggs	2744	14.1	4.486	1.9	146.304	13.3	2.010
Freshwater Fish	2761	0.1	*25.430*	16.7	*125.450*	3.2	*2.080*
Grapefruit	2613	4.0	*3.100*	0.4	*15.290*	2.4	*0.250*
Grapes (excl wine)	2620	61.9	*10.110*	0.3	*49.870*	12.0	*0.830*
Maize	2514	183.2	0.724	10.3	136.500	310.8	0.253
Milk	2848	204.0	1.508	26.4	34.970	268.5	0.752
Molluscs	2767	0.8	*47.690*	0.0	*235.220*	3.5	*3.900*
Mutton/Goat Meat	2732	1.3	*3.340*	1.3	*16.470*	1.0	*1.805*
Nuts	2551	0.4	*11.140*	0.9	*54.560*	4.5	*0.910*
Oats	2516	10.5	0.600	0.0	18.820	32.9	0.205
Onions	2602	12.8	*3.820*	8.6	*16.780*	10.2	*0.353*
Oranges, Mandarines	2611	23.8	0.464	0.9	16.940	41.9	0.280
Palm Oil	2577	0.0	*16.850*	6.4	*83.110*	1.7	*1.380*
Peas	2547	0.1	*6.990*	1.5	*34.500*	9.5	*0.489*
Pelagic Fish	2763	1.7	*30.200*	0.8	*148.970*	13.2	*2.470*
Pig Meat	2733	9.0	5.690	0.0	112.900	28.8	1.415
Pineapples	2618	1.0	*10.810*	1.4	*53.320*	4.6	*0.880*
Potatoes	2531	44.3	0.402	54.2	7.480	94.7	0.272
Poultry Meat	2734	35.3	4.472	1.4	2.590	37.1	2.168
Pulses, Other	2549	0.4	*8.760*	2.3	*25.500*	12.0	*0.493*
Rape and Mustard Oil	2574	0.0	*14.620*	0.7	*72.130*	17.3	*1.200*
Rice (Milled Equivalent)	2805	10.0	0.986	195.4	28.098	11.1	0.420
Sorghum	2518	64.1	0.702	0.0	18.500	0.1	0.310
Soybean Oil	2571	59.2	*19.390*	2.8	*95.650*	7.6	*1.590*
Soybeans	2555	954.3	1.247	1.3	37.580	54.8	0.449
Sugar (Raw Equivalent)	2542	41.7	*8.900*	9.0	*43.910*	34.0	*0.730*
Sunflower Seed Oil	2573	11.4	*15.260*	0.0	*75.270*	1.2	*1.250*
Sweet potatoes	2533	9.6	*6.580*	1.9	*12.420*	1.5	*0.540*
Tomatoes	2601	18.9	0.822	1.7	13.180	22.5	0.165
Vegetables, Other	2605	47.7	*14.160*	18.0	*12.530*	88.5	*1.794*
Wheat	2511	131.0	0.904	22.2	19.530	145.0	0.224

Source: FAOSTAT, quantities [27] and prices [28] (Prices in italics are extrapolated from values in other countries).

**Table 2:** Typical food item energy, protein, and selected nutrients (per kg).

	Energy	Protein	Vit C	Thiamin	Riboflavin	Niacin	Folate	Ca	Fe
	MJ	g	mg	mg	mg	mg	μg	mg	mg
Apples	1.09	2	100	0.4	0.2	1	40	30	1
Bananas	2.7	8	70	0.3	0.4	5	90	40	2
Barley	14	120	0	4.5	1.8	61	1300	550	65
Beans, green	6.15	130	290	4.4	1.8	16.5	1650	1970	35.5
Bovine Meat	25.26	119	0	0.7	2.4	52	100	140	14
Butter, Ghee	36.93	0	0	0	0	0	0	0	2
Cassava	6.7	14	206	0.9	5	8.5	270	160	2.7
Cocoa Beans	6	8	0	0.8	0.3	8.5	100	200	20
Crustaceans	1.05	40	0	0.2	0.3	5	40	60	3
Dates	9.7	28	120	0.5	0.7	1	160	30	7
Demersal Fish	3.86	179	0	3	1	32	100	220	3
Eggs	6.12	125	0	0.9	4.7	1	500	570	19
Freshwater Fish	3.75	155	0	1.3	0.8	2.8	0	240	7
Grapefruit	0.86	5	240	0.3	0.1	2	180	160	1
Grapes (excl wine)	2.44	4	30	0.5	0.1	2	20	130	3
Maize	15.28	94	0	3.9	2	36.3	190	70	27.1
Milk	2.75	32	1	0.3	1.7	1	60	1150	0.6
Molluscs	1.11	52	0	0	0.8	3	0	590	23
Mutton/Goat Meat	15.9	147	0	0.9	1.6	39	30	70	12
Nuts	25.2	229	0	2.2	2.2	99	540	780	21
Oats	15.9	112	0	9	0.9	8	600	520	38
Onions	1.5	12	50	0.9	0	3	9	250	3
Oranges, Mandarines	1.35	7	200	0.8	0.1	2	120	170	5
Palm Oil	40	0	0	0	0	0	0	0	0
Peas	3.44	69	240	7.4	0.2	25	620	210	28
Pelagic Fish	5.86	325	0	0	0	0	0	220	18
Pig Meat	16.5	211	0	5.3	1.1	42	40	110	10
Pineapples	1.76	4	120	0.8	0.3	3	50	180	2
Potatoes	3.18	21	1.5	0.15	0.2	4	350	50	4
Poultry Meat	5.08	205	0	1	1.6	78	120	100	70
Pulses, Other	12	220	0	4	2.5	20	1000	700	100
Rape and Mustard Oil	40	0	0	0	0	0	0	0	0
Rice (Milled Equivalent)	15.2	67	0	5.9	0.7	5.3	490	100	14
Sorghum	13.77	106	0	3.3	1	37	200	130	34
Soybean Oil	40	0	0	0	0	0	0	0	0
Soybeans	15.5	359	0	6.1	2.7	22	3700	2400	97
Sugar (Raw Equivalent)	16.8	0	0	0	0	0	0	0	0
Sunflower Seed Oil	40	0	0	0	0	0	0	0	0
Sweet potatoes	3.72	12	230	1.7	0	5	170	240	7
Tomatoes	0.73	7	170	0.9	0.1	10	170	70	5
Vegetables, Other	1.8	33	130	1.2	0.9	8	520	350	8
Wheat	14	120	0	4.5	1.8	61	510	550	65

Sources: Southgate (1991) [30], USDA (2006) [26].

**Table 3:** Example of national daily intake target.

Energy	Protein	Vit C	Thiamin	Riboflavin	Niacin	Folate	Ca	Fe
MJ	g	mg	mg	mg	mg	μg	mg	mg
10	55	40	1	1.3	14.5	200	700	14

**Table 4:** Case studies: Food intake for sufficient energy.

	SET 1		SET 2		SET 3	
	g/day	LCU/day	g/day	LCU/day	g/day	LCU/day
Argentina						
Wheat	55.0	0.050	89.2	0.081	55.0	0.050
Other Cereal	84.4	0.061	10.1	0.007	85.0	0.062
Rice	8.4	0.008	61.4	0.061	8.0	0.008
Potatoes	18.6	0.007	99.3	0.040	0	0
Green Veg	4.2	0.016	108.0	0.413	0	0
Tomato	8.4	0.007	67.5	0.056	234.1	0.192
Pulses	428.1	0.534	214.8	0.268	590.0	0.736
Fruits	50.9	0.026	306.8	0.155	0	0
Milk Products	85.6	0.129	368.2	0.555	203.1	0.306
Beef Meat	27.9	0.218	28.5	0.223	0	0
Poultry Meat	14.8	0.066	112.2	0.502	0	0
White Fish	1.6	0.042	8.8	0.223	0	0
Shell Fish	0.5	0.022	4.4	0.209	0	0
Nuts	0.2	0.002	16.0	0.178	0	0
Eggs	5.9	0.027	9.2	0.041	0	0
Vegetable Oil	29.7	0.453	33.7	0.515	0	0
Sugar	17.9	0.159	2.6	0.023	0	0
cost/cap/day (Argentine peso)		1.826		3.549		1.354
**Bangladesh**						
Wheat	45.7	0.893	89.2	1.742	45	0.88
Other Cereal	21.4	2.922	10.1	1.376	20	2.73
Rice	407.4	11.447	61.4	1.724	410	11.52
Potatoes	112.7	0.843	99.3	0.743	0	0
Green Veg	12.5	0.156	108.0	1.354	304	3.81
Tomato	5.2	0.068	67.5	0.890	0	0
Pulses	64.4	1.643	214.8	5.477	0	0
Fruits	19.3	0.679	306.8	10.824	0	0
Milk Products	54.9	1.919	368.2	12.875	439	15.36
Beef Meat	5.4	0.610	28.5	3.216	0	0
Poultry Meat	2.9	0.008	112.2	0.291	212	0.55
White Fish	36.0	4.511	8.8	1.100	0	0
Shell Fish	2.5	0.587	4.4	1.031	0	0
Nuts	1.9	0.102	16.0	0.873	0	0
Eggs	3.9	0.578	9.2	1.347	0	0
Vegetable Oil	20.9	1.510	33.7	2.434	0	0
Sugar	18.7	0.821	2.6	0.115	0	0
cost/cap/day (Bangladeshi Taka)		29.298		47.411		34.852
**Canada**						
Wheat	142.9	0.032	89.2	0.020	140	0.031
Other Cereal	32.5	0.008	10.0	0.003	30	0.008
Rice	44.3	0.019	61.4	0.026	40	0.017
Potatoes	93.3	0.025	99.3	0.027	0	0
Green Veg	10.8	0.005	108.1	0.053	0	0
Tomato	23.6	0.004	67.5	0.011	232	0.038
Pulses	162.6	0.073	214.9	0.096	29	0.013
Fruits	96.0	0.038	307.0	0.120	0	0
Milk Products	264.5	0.199	368.4	0.277	501	0.377
Beef Meat	58.7	0.127	28.5	0.062	0	0
Poultry Meat	36.6	0.079	112.3	0.243	43	0.092
White Fish	8.2	0.017	8.8	0.018	0	0
Shell Fish	9.2	0.036	4.4	0.017	0	0
Nuts	4.4	0.004	16.0	0.015	0	0
Eggs	13.1	0.026	9.2	0.019	0	0
Vegetable Oil	30.2	0.036	33.8	0.041	122	0.146
Sugar	36.1	0.026	2.6	0.002	0	0
cost/cap/day (Canadian Dollar)		0.755		1.049		0.722

**Table 5:** Case studies: Nutrient intake achieved.

	Energy	Protein	Vit C	Thiamin	Riboflavin	Niacin	Folate	Ca	Fe
	MJ	g	mg	mg	mg	mg	μg	mg	mg
Argentina									
SET 1	**10.0**	120.1	*7.2*	2.44	1.63	18.0	501	*449*	50.6
SET 2	**10.0**	114.3	56.7	2.29	1.91	24.9	459	724	39.5
SET 3	**10.0**	153.1	**40.0**	3.26	2.12	20.8	690	**700**	66.3
**Bangladesh**									
SET 1	**10.0**	61.4	*4.7*	3.07	*0.77*	*8.5*	346	*201*	17.0
SET 2	**10.0**	114.3	56.7	2.29	1.91	24.9	459	724	39.5
SET 3	**10.0**	102.3	**40.0**	3.41	1.77	25.0	438	**700**	26.7
**Canada**									
SET 1	**10.0**	88.9	15.4	1.96	1.54	20.8	345	546	31.9
SET 2	**10.0**	114.4	56.8	2.29	1.91	24.9	459	724	39.5
SET 3	**10.0**	**55.0**	**40.0**	1.50	1.35	16.6	**200**	**700**	17.8

Intake at target in **bold**, below target in *italic*.
